# Unveiling the Gut Microbiome: How Junk Food Impacts the Gut

**DOI:** 10.7759/cureus.49179

**Published:** 2023-11-21

**Authors:** Sania S Shah, Obaid Noman, Neha Jaiswal

**Affiliations:** 1 Microbiology, Datta Meghe Medical College, Datta Meghe Institute of Higher Education and Research (DU), Wardha, IND; 2 Pathology, Datta Meghe Medical College, Datta Meghe Institute of Higher Education and Research (DU), Wardha, IND

**Keywords:** healthy food, chronic disease, diet, immunity, disease, health, dysbiosis, microbiota, gut microbiome

## Abstract

The human gut microbiome, a complex community of microorganisms, profoundly influences human health and disease. Bacteroidetes and Firmicutes make up the majority of the normal human gut microbiota. These microorganisms wield considerable influence over our physiological functions, impacting both our well-being and our susceptibility to disease. The surge of interest in the gut microbiome over the past decade has been remarkable. Once overlooked, the gastrointestinal tract’s microbiota has gained recognition for its significance in maintaining optimal health. The food industry has capitalized on this, flooding the market with “probiotic” and “fermented” products. This article aims to provide a critical review of the current literature on the gut microbiome and its significance in human health, with a particular focus on the impact of dietary choices, especially junk food, on the composition and function of the gut microbiota. Microbes possess the remarkable ability to unlock nutrients from otherwise indigestible substances. The gut microbiome of individuals who consume healthy foods and those who prefer junk food varies significantly. Healthy diets promote a diverse and beneficial gut microbiome, while junk food consumption often leads to a less diverse microbiome with negative consequences for health.

## Introduction and background

The human body hosts a diverse array of microorganisms, including bacteria, archaea, viruses, and eukaryotic microbes, collectively referred to as the human microbiome. Over 100 trillion microbial cells reside in our gut, where they form a complex ecosystem that affects human physiology, metabolism, nutrition, and immune function [1]. Bacteroidetes and Firmicutes make up the majority of the normal human gut microbiota. These microorganisms wield considerable influence over our physiological functions, impacting both our well-being and our susceptibility to disease [2]. They play pivotal roles in metabolism, protection against pathogens, and immune system education and, by extension, affect a wide range of bodily functions. Technological advancements have propelled the study of the human microbiome by enabling culture-independent analyses, a breakthrough in understanding these complex communities. Advancements in characterizing the microbiome’s structure have paved the way for investigating its functional interactions with the host. Understanding these functions is pivotal in comprehending the microbiome’s role in human health and disease [3].

The surge of interest in the gut microbiome over the past decade has been remarkable. Once overlooked, the gastrointestinal tract’s microbiota has gained recognition for its significance in maintaining optimal health. The food industry has capitalized on this, flooding the market with “probiotic” and “fermented” products. This newfound attention, however, has led to confusion due to the burgeoning data that leaves many questions unanswered [4]. The concept of influencing gut health through microorganisms isn’t new. Early in the 20th century, Élie Metchnikoff associated the longevity of rural Bulgarians with their consumption of fermented milk products [5]. He proposed that these products, rich in lactic acid bacteria, contributed to their extended lifespans. Metchnikoff’s work laid the foundation for understanding how healthy bacteria could replace harmful ones, a concept that earned him a Nobel Prize. However, the discovery of antibiotics shifted the focus away from bacterial therapies. As antibiotic resistance becomes a growing concern, researchers are revisiting bacterial interventions, especially with the advent of advanced molecular techniques [5,6]. In the realm of microbiome research, significant strides have been made in unraveling the mysteries of our body’s intricate microbial communities. Technological advancements have empowered the human race to explore the complex world of bacteria beyond traditional cultivation methods. Techniques such as 16S rRNA gene sequencing and metagenomic analysis have allowed us to delve into both the identity and potential functionality of these microorganisms [7]. This article aims to provide a critical review of the current literature on the gut microbiome and its significance in human health, with a particular focus on the impact of dietary choices, especially junk food, on the composition and function of the gut microbiota.

## Review

Methodology

**Figure 1:**
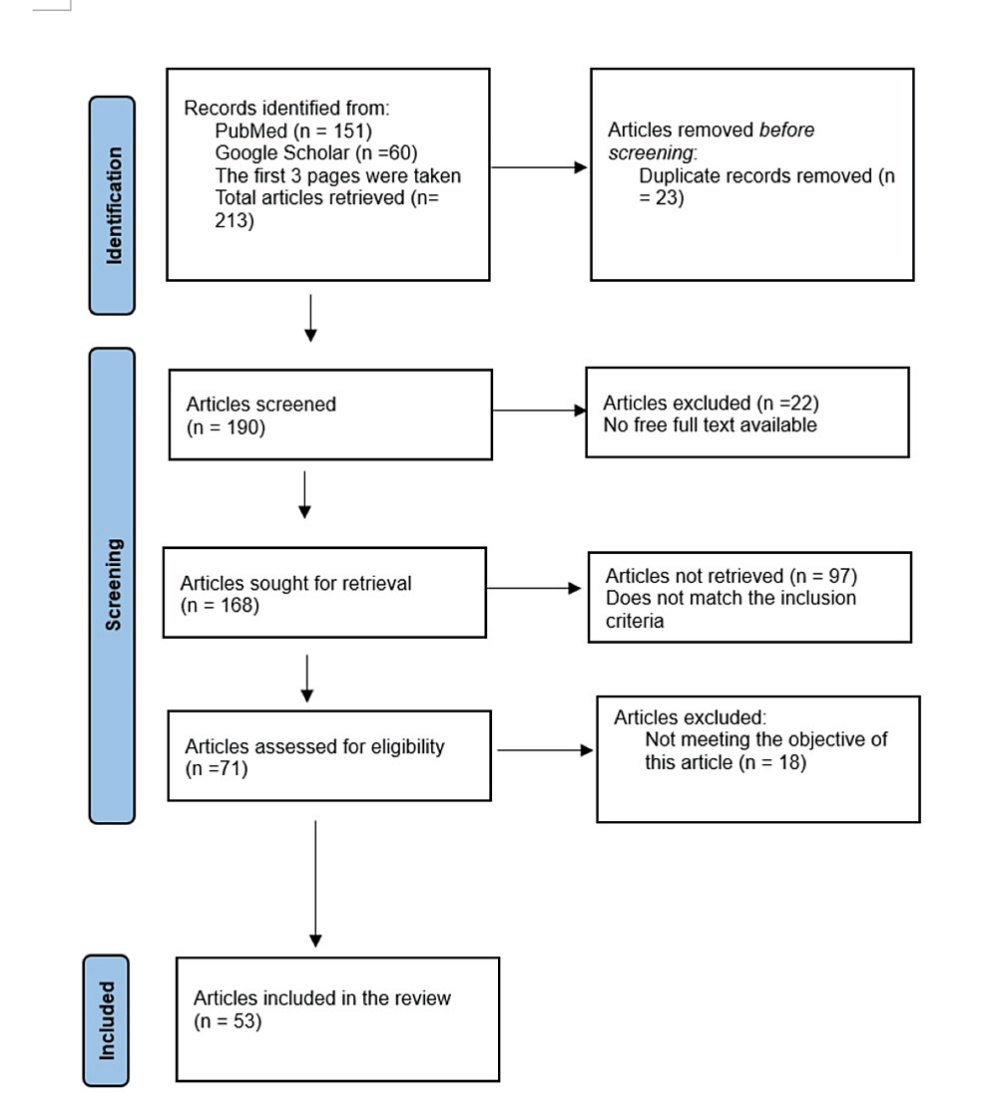
PRISMA flow diagram n, number of studies; PRISMA, preferred reporting items for systematic reviews and meta-analyses

To conduct a comprehensive literature search for a review article, we used the following databases: PubMed and Google Scholar. We searched for articles using the following search terms: (gut microbiome) OR (Gut microbiome) AND (Microbiota) OR (microbiota) AND (healthy food) OR ( healthy diet) OR (nutritional food) AND (chronic disease) OR (long-term disease) AND (immunity) AND (dysbiosis). We applied the following inclusion criteria for the final review: (1) review articles, (2) English language, (3) peer-reviewed, (4) relevant to the topic, and (5) full-text available (Figure 1) [8].

Advancements in microbiome study

The advancement of technology has heralded a remarkable era in microbiome research, providing us with unprecedented tools to unravel the intricacies of microbial communities without the need to culture them in a lab. This scientific progress has been instrumental in exploring the world of microbes that inhabit the human body.

16S rRNA Gene Sequencing

Gene sequencing technique has been a linchpin in microbiome research. It enables scientists to zero in on specific bacterial populations by sequencing the variable regions of the 16S rRNA gene, a genetic marker that is present in all bacteria. It’s similar to identifying individual species by their unique DNA fingerprints but in the world of bacteria [7]. This approach has been instrumental in characterizing the composition and diversity of microbial communities residing in the human microbiome. It has provided a taxonomic roadmap, allowing researchers to identify and classify various microbial players, leading to a better understanding of who inhabits our microbiome [9].

Metagenomic Analysis

Metagenomic analysis takes the exploration to a more comprehensive level by scrutinizing the entirety of microbial DNA present in a given sample. This expansive approach goes beyond mere identification and uncovers the vast genetic potential encoded in the microbial genomes. It’s similar to reading the entire library of genetic information within the microbiome [10]. By doing so, researchers not only identify the microbial residents but also gain insights into their functional potential. This helps us understand what these microorganisms are capable of genetically and, importantly, how these capabilities may influence human health [11].

Meta-Transcriptomics

Meta-transcriptomics method delves into RNA, the dynamic molecule that reflects active gene expression within the microbiome. While genes provide a blueprint, RNA reveals the current construction project [12]. Meta-transcriptomics allows scientists to understand which genes are actively transcribed and which proteins are being produced by the microbial community. It provides a real-time snapshot of the activities within the microbiome, shedding light on how microorganisms engage with their environment, including their host [13].

Meta-Proteomics

Meta-proteomics goes even further by focusing on the proteins expressed by the microbiome. Proteins are the workhorses of biological processes, and by analyzing them, researchers gain insights into the functional aspects of the microbial community [14]. This method helps us understand how microorganisms interact with each other and with the human host. It unveils the active machinery within the microbiome and provides a window into the functions and activities of these tiny inhabitants [15].

Metabolomics

Metabolomics provides a glimpse of the small molecules (metabolites) produced by the microbiome. These metabolites include essential nutrients, signaling molecules, and byproducts of microbial metabolism [16]. Metabolites are critical players in host-microbe interactions, influencing various aspects of human health, from immune responses to metabolic processes. This approach reveals how the microbiome can impact the overall physiology of the host, offering insights into its far-reaching effects on health and disease (Table 1) [17].

**Table 1:** Advancement in microbiome studies

Advancement	Description
16S rRNA gene sequencing	Identifying individual species by their unique DNA fingerprints, taxonomic roadmap [7,8]
Metagenomic analysis	Identifying and uncovering the vast genetic potential encoded in the microbial genomes [10]
Meta-transcriptomics	Reflecting active gene expression within the microbiome [12]
Meta-proteomics	Microorganisms interact with each other and with the human host [14]
Metabolomics	Impact of microbiome on host [16]

Data accumulation on human microbiome

The MetaHIT study, in particular, stands out for its ambitious exploration of the genetic content of fecal microbial genes. This initiative delved deep into the microbiome’s genetic makeup, analyzing over 3 million fecal microbial genes [18]. Extensive genomic profiling has provided a detailed and intricate insight into the genetic diversity of these microorganisms. By pooling metagenomic data from various sources and samples, these projects have successfully compiled an extensive gene catalog comprising approximately 9.8 million microbial genes [19].

The sheer magnitude of this genetic data reflects the astonishing diversity and variability within these microbial communities. Each sample under scrutiny has, on average, been found to contain around 750,000 genes, highlighting the complexity of the microbiome and the vast genetic potential it holds [20]. This wealth of information has opened doors to exploring the functional aspects of these genes, shedding light on the metabolic capabilities and potential contributions of the microbiome to human health [21].

The thoroughness and scale of these data collection efforts represent a significant step forward in microbiome research. It provides a foundation for studying the microbiome’s role in various health conditions, including obesity, metabolic disorders, and gastrointestinal diseases [22]. It not only enhances our understanding of the microbiome’s genetic diversity but also underscores the intricate relationship between these microbial communities and the human host. The continued analysis of such extensive genetic data holds great promise for advancing personalized medicine, dietary interventions, and the development of novel therapies based on microbiome insights [23].

Microbiome and diseases

Microbes possess the remarkable ability to unlock nutrients from otherwise indigestible substances. For example, certain species of *Bacteroides* engage in the digestion of xyloglucans, which has significant implications for our dietary choices. Moreover, microbiota generates short-chain fatty acids (SCFA) from dietary fibers as an essential energy source [24]. The complex interplay that takes place between our bodies and the microorganisms that live within them is incredibly fascinating. The concept of dysbiosis, wherein the balance of our microbial companions shifts, has captured considerable attention [25]. Yet, navigating the labyrinthine relationship between these changes and diseases proves a challenging puzzle. The question of what causes what remains enigmatic, with microbiota changes often a response to diseases or interventions like antibiotics. This exploration gains further complexity as we delve into the roles of microbiota in specific conditions, such as rheumatoid arthritis, cardiovascular disease, obesity, colorectal cancer, and diabetes, where the tendrils of microbial influence are still being untangled [26]. Gut microbiota plays a significant role in heart health, influencing cardiovascular disease through dietary phosphatidylcholine [27]. Treatments for irritable bowel syndrome (IBS) include dietary changes, probiotics, and antibiotics [28]. The microbiota-gut-brain axis connects gut changes with central nervous system symptoms [29]. Clostridium difficile infection (CDI) is rooted in gut microbiota, and microbiota-based therapies like fecal microbiota transplant (FMT) can prevent recurrent CDI [30]. Inflammatory bowel disease (IBD) is a complex disease where environmental and genetic factors intersect, with microbial dysbiosis sometimes acting as both the cause and consequence of inflammation [31].

The alterations in brain-gut microbiota interactions are believed to be involved in the pathogenesis of brain disorders like IBS and functional gastrointestinal disorders. These alterations are also linked to brain disorders like autism spectrum disorders, Parkinson’s disease, mood and affect disorders, and chronic pain [32]. The gut microbiota and its metabolites modulate GI functions, behaviors, and brain processes, including stress responsiveness, emotional behavior, pain modulation, ingestive behavior, and brain biochemistry [33].

Gut microbiome composition in junk food consumers

The human gut is a dynamic community that interacts intimately with human physiology and has been implicated in a wide range of health outcomes, from metabolism and immunity to mental well-being [34]. Healthy food is rich in fiber, diverse, high in nutrients, low in added sugars, and balanced in carbohydrates, proteins, and lipids, promoting health and reducing the risk of metabolic diseases [35]. Junk food is high in saturated fats and sugars, with processed ingredients, additives, preservatives, and synthetic flavors. It lacks fiber, causes digestive problems, and lacks essential nutrients, leading to weight gain and metabolic disruption (Table 2) [36].

**Table 2:** Gut microbiome composition in junk food consumers [37-40]

Composition	Descriptions
Reduced microbiome diversity	Junk food diets are often associated with a reduction in the diversity of the gut microbiome. This means that there are fewer different types of microorganisms in the gut, which can have a negative impact on overall health.
Shift toward harmful bacteria	Junk food consumption is linked to an increase in potentially harmful bacteria in the gut. These microorganisms can contribute to various health issues, including inflammation and metabolic problems.
Lower levels of beneficial bacteria	Healthy diets that are rich in fiber and nutrients promote the growth of beneficial bacteria in the gut, which can have a positive impact on digestion and overall health. In contrast, junk food diets typically lack these beneficial nutrients, leading to lower levels of helpful microorganisms.
Imbalanced microbiome	The gut microbiome of junk food consumers often lacks the balance that is crucial for maintaining a healthy digestive system. This imbalance can lead to digestive problems and an increased risk of metabolic disorders.

Diet and gut microbiome composition

The gut microbiome is highly responsive to dietary inputs, and research has consistently demonstrated that what we eat can significantly impact its composition (Table 3) [41]. 

**Table 3:** Changes in gut composition in relation to food consumption [36,40,42] IGF, insulin-like growth factor; IBD, inflammatory bowel disease; SCFA, short-chain fatty acids

Components	Composition	Impact
Protein	Increased whey and pea protein intake	Increases beneficial *Lactobacillus* and *Bifidobacterium*; reduces harmful *Clostridium* and *Bacteroides*
	Increased animal-based protein	Increases bile-tolerant anaerobes like *Bacteroides*, *Alistipes*, and *Bilophila*
	Increased total protein	Reduces certain beneficial bacteria and butyrate production; increases risk of IBD, higher levels of IGF-1: linked to cancer and diabetes risk
Fats	High-fat Western diets	Increases anaerobic microflora and *Bacteroides* counts
	Low-fat diet	Increases *Bifidobacterium*; reduces glucose and total cholesterol
	High saturated fat diet	Increases *Faecalibacterium prausnitzii*
	High monounsaturated fat intake	Reduces overall bacterial load and plasma cholesterol
Digestible carbohydrates	High glucose, fructose, and sucrose Intake	Increases *Bifidobacteria*; reduces *Bacteroides*
	Lactose supplementation	Reduces *Clostridia* species; increases beneficial SCFA concentration.
Non-digestible carbohydrates (fiber)	Probiotics-fermented food (cultured milk products, yogurt)	Increases total bacterial load and beneficial bacteria like *Bifidobacteria* and *Lactobacillus*; reduces enteropathogens like* Escherichia coli* and *Helicobacter pylori*
	Polyphenols (fruits, seeds, vegetables, tea, cocoa, and wine): catechins, flavanols, and phenolic acids	Increases *Bifidobacterium* and *Lactobacillus* and antibacterial activity against pathogens like *Staphylococcus aureus*, *Salmonella typhimurium*, pathogenic *Clostridium* species
	Prebiotics: soybeans, insulins, whole grains, and oligosaccharides	Increases *Bifidobacteria*, lactic acid bacteria, *Ruminococcus*, and *Eubacterium rectale*; reduces *Clostridium* and *Enterococcus*

The gut microbiome of individuals who consume healthy foods and those who prefer junk food varies significantly. Healthy eaters have a more diverse gut microbiome, with beneficial bacteria like Bifidobacterium and Lactobacillus being more prevalent [42]. They also consume high-fiber foods, which provide prebiotics that support the growth of good bacteria in the stomach. In contrast, junk food eaters have a reduced diversity, leading to negative changes such as increased harmful bacteria growth and inflammation [43].

Healthy eaters and their gut microbiome

Individuals who adhere to a diet centered around nutritious, whole foods typically enhance a gut microbiome that reflects the positive impact of their dietary choices. Several key characteristics distinguish this group (Table 4).

**Table 4:** Healthy eaters and their gut microbiome [44-46] SCFA, short-chain fatty acids

Healthy eater's gut microbiome	Impact
Microbiome diversity	Healthy eaters tend to exhibit a higher diversity in their gut microbiome. This means that they host a wider array of microbial species in their digestive tracts. This diversity is linked to improved gut health and resilience.
Beneficial bacteria abundance	The gut microbiome of healthy eaters is often characterized by a higher prevalence of beneficial bacteria, such as *Bifidobacterium* and *Lactobacillus*. These microorganisms play essential roles in digestion and the production of beneficial compounds like SCFAs, supporting the gut's protective barrier.
High-fiber diet	Healthy food enthusiasts consume diets rich in fiber, which are found in foods like fruits, vegetables, and whole grains. Fiber acts as a prebiotic, providing nourishment for the growth of beneficial bacteria in the gut. This dietary component promotes the proliferation of these helpful microorganisms.

Junk food consumers and their gut microbiome

In complete contrast, individuals who favor diets predominantly composed of junk food exhibit a gut microbiome with distinct characteristics, often associated with negative consequences for health (Table 5).

**Table 5:** Junk food consumers and their gut microbiome [23,47,48]

Junk food consumer's gut microbiome	Impact
Reduced diversity	Junk food diets are frequently linked to a reduction in the diversity of the gut microbiome. This means that there are fewer types of microorganisms present, which can reduce the microbiome's resilience and functionality.
Harmful bacteria proliferation	Junk food consumption is often associated with the increased presence of potentially harmful bacteria in the gut. These microorganisms can contribute to various health issues, including inflammation and metabolic problems.
Imbalanced microbiome	The gut microbiome of junk food consumers often lacks the balance that is crucial for maintaining a healthy digestive system. This imbalance can lead to digestive problems and an increased risk of metabolic disorders.

Healthy eaters have a rich diversity of beneficial bacteria, such as bifidobacteria and lactobacilli, which are essential for digestion, production of SCFA, and gut wall reinforcement [49]. Junk food, on the other hand, encourages the expansion of harmful microorganisms like Firmicutes, linked to inflammation and obesity. SCFAs play a crucial role in maintaining gut health by acting as an energy source for gut bacteria, maintaining gut barrier function, promoting immune modulation, regulating gut pH, influencing appetite and metabolism, anti-inflammatory effects, increasing mucus formation, and protecting against pathogens [50].

A robust gut barrier and decreased systemic inflammation are linked to a diet high in nutritious foods, while diets high in junk food can result in chronic inflammation and weakened gut defenses, leading to systemic health problems [51]. The gut microbiome is also crucial in controlling metabolism, with healthy individuals having microbiomes that support improved cholesterol and glucose metabolism [51]. Understanding these differences in gut microbiome composition can lead to dietary interventions to promote gut health and reduce related health risks [52,53]. These include fiber-rich diets, prebiotics, probiotics, behavioral interventions like education and behavioral therapy, and personalized nutrition based on individual gut microbiota composition [54].

## Conclusions

Microbiome research has made significant progress in understanding our body’s microbial communities, using techniques like 16S rRNA gene sequencing and metagenomic analysis. Large-scale initiatives like MetaHIT and HMP have provided a vast repository of data on the human microbiome’s diversity and genetic makeup. The gut microbiome is shaped by dietary choices, with healthy eaters having a diverse microbiome with beneficial bacteria, while junk food consumption leads to reduced diversity and an overabundance of pathogenic species. These dietary disparities impact inflammation, metabolic health, and overall well-being. Understanding the relationship between diet and gut microbiome can help promote gut health and prevent chronic diseases. Future research should continue to refine strategies for improving gut microbiome composition.
